# Genetic improvement of a synthetic microbiota: a step further?

**DOI:** 10.3389/fmicb.2025.1619874

**Published:** 2025-07-16

**Authors:** Marcel Martínez-Porchas, Diana Medina-Félix, Francisco Vargas-Albores, Estefanía Garibay-Valdez, Yuniel Méndez-Martínez, Luis Rafael Martínez-Córdova, Angel Martin Ortiz-Estrada

**Affiliations:** ^1^Centro de Investigación en Alimentación y Desarrollo, A. C. Biología de Organismos Acuáticos, Hermosillo, Sonora, Mexico; ^2^Departamento de Ecología, Universidad Estatal de Sonora, Hermosillo, Sonora, Mexico; ^3^Facultad de Ciencias Pecuarias y Biológicas, Universidad Técnica Estatal de Quevedo, Quevedo, Los Ríos, Ecuador; ^4^Departamento de Investigaciones Científicas y Tecnológicas de la Universidad de Sonora, Universidad de Sonora, Hermosillo, Sonora, Mexico; ^5^Universidad Estatal de Sonora, Navojoa, Sonora, Mexico

**Keywords:** gut microbiota, synthetic microbiota, genetically modified microbiota, biotechnology, genetically modified microorganism (GMM), gut microbiota therapeutics

## Introduction

Synthetic gut microbiota refers to *in vitro* assembled microbial consortia designed to mimic a particular microbial composition and functional characteristics, as has been done in humans and other animal species. Although reduced in member numbers, the synthetic microbial community should reflect most of the corresponding set of microbes living in the intestinal cavity, allowing for studying microbial behavior and interactions within a controlled environment (Li et al., 2024). Such an approach, combined with gnotobiology (research involving animals raised in the absence of microorganisms or the presence of known microbial strains or communities), has provided most of the information about the biological relevance of the gut microbiota (Mooser et al., 2018). Briefly, gnotobiology allows the isolation and analysis of specific effects on individual groups of microbes on host health, metabolism, and immunity, which is impossible in naturally colonized systems.

Beyond understanding the biological role of the gut microbiota, a synthetic microbiota can be used for healthcare purposes, starting with recolonization after dysbiosis caused by antibiotics, diseases, or other factors. However, this approach can go even further, becoming a therapeutic measure focused on specific objectives, performing special biochemical activities, or regulating particular host functions via neural or endocrine pathways. In this regard, synthetic biology can provide tools to manipulate microbes genetically (Nazir et al., 2024; Xin and Qiao, 2025), enabling some to perform desired functions. For instance, engineered bacteria are used as living therapeutic agents for delivering into the intestine diverse biomolecules, such as bacteriocins, enzymes, cytokines, allergens, and bioactive peptides (Romero-Luna et al., 2022). Engineered bacteria may improve communication with the host by modulating specific biological systems.

Genetically modified probiotics demonstrate the technical feasibility of this proposal (Mazhar et al., 2020); however, ethical, regulatory, and technical issues constitute significant challenges to this approach. Regarding technical issues, not all bacteria are genetically manipulable, at least with the current methods. In this paper, we provide an opinion on the potential of developing genetically modified synthetic microbiota for use in healthcare.

## Engineering bacteria and some applications

A genetically modified bacterium (GMB) is described as capable of effectively producing heterologous (foreign) proteins or molecular compounds for a particular function following genetic modification (Liu et al., 2022). In this regard, three general types of genetic modification can be listed: insertion, deletion, and gene replacement.

Several recombination technologies can be used for genetic manipulation, including conventional approaches such as bacterial artificial chromosome (BAC), conjugate transfer, transposition recombination, and phage infection. A BAC is a large DNA fragment, usually 100 to 300 kb, designed for insertion into bacteria to be propagated as a circular artificial chromosome (Shizuya and Kouros-Mehr, 2001). However, this technique is limited to *Escherichia coli* as the chassis, and it involves transferring DNA between bacteria through direct contact via conjugative pili (Sana et al., 2014). Conjugation is a widely preserved DNA transfer process found in both Gram-negative and Gram-positive bacteria, providing a possibility of genetically engineering commensal gut bacteria. On the other hand, transposition recombination systems employ relatively straightforward mechanisms involving transposase and site-specific recombinase enzymes that facilitate the essential processes of DNA breakage and subsequent joining reactions (Hallet and Sherratt, 1997); these cut-and-paste mechanisms in conserved DNA fractions can create a range of intricate DNA rearrangements. Finally, phage engineering involves using virus-containing genetically engineered phages introduced into a host bacterial cell to kill it or alter its gene expression, thus manipulating its functionality.

While these approaches have offered valuable insights into microbial engineering, they are often time-consuming, limited to a few microbes, and not adaptable for creating synthetic microbiota. Next-generation genetic editing tools like zinc finger nucleases (ZFN; Porteus and Carroll, 2005), transcription activator-like effector nucleases (TALEN; Sun and Zhao, 2013), and CRISPR-Cas9 (Jiang and Doudna, 2017) have advanced the field further. However, ZFN and TALEN are costly, time-intensive, and can introduce non-specific mutations.

CRISPR-Cas, on the other hand, is an efficient and flexible method for engineering bacteria, utilizing a bacterial defense system that protects against viral DNA invasions. It consists of three phases: recognition, cleavage, and repair. In the recognition phase, the Cas9 protein binds to a single guide RNA (sgRNA) with a 20-base pair sequence complementary to the target gene, positioning it near the protospacer-adjacent motif. This helps to guide the Cas9 protein to the target gene. Introducing the CRISPR-Cas9 complex into the cell results in the formation of double-strand breaks (DSBs) at the specific genomic location (Allemailem et al., 2024). Considering that most Archaea and at least half of the known bacteria have some variant of the CRISPR-Cas defense system (Goh and Barrangou, 2019), this is perhaps the most adequate technology so far to genetically improve synthetic gut microbiota.

Although six CRISPR-Cas types and 29 subtypes have been identified so far, the conserved protospacer-adjacent motif sequences in all bacteria allow Cas nucleases to cleavage in the target DNA and constitute a significant factor to consider when designing target-specific guide RNAs (Goh and Barrangou, 2019). One advantage of CRISPR-Cas over integrative methods using plasmids is the lower risk of losing the incorporated genetic material if successful.

Recent advances have demonstrated the feasibility of genetically manipulating gut microbiota using CRISPR-based technologies for therapeutic purposes. A recent study modified the probiotic yeast *Saccharomyces boulardii* to biosynthesize β-carotene (vitamin A precursor) directly within the intestines of mice, demonstrating the feasibility of using live microorganisms for localized and sustained micronutrient production in the gastrointestinal tract (Durmusoglu et al., 2021). A genetically modified *Lactococcus lactis* strain expressing the human enzyme ADH1B, which enhanced the metabolic conversion of alcohol to acetate, resulting in lower blood acetaldehyde levels and reduced liver damage in alcohol-exposed animal models (Jiang et al., 2023). To improve biosafety, synthetic gut microorganisms have been equipped with CRISPR-based kill switches that obliterate themselves in particular environmental situations, guaranteeing regulated persistence within the host (Chan et al., 2016). Collectively, these studies underscore the promise of CRISPR in engineering gut-resident microbes for precision medicine and disease prevention. However, information about the topic is limited in its early development.

Practical applications in this field are limited and reduced to the modification of single strains, with notable examples, including the engineering of *Escherichia coli* to detect and respond to gut inflammation, serving as a biosensor for inflammatory bowel disease (IBD). For instance, Riglar et al. (2017) created a synthetic genetic circuit in *E. coli* that records inflammatory signals in the gut, allowing for non-invasive monitoring of disease states in mice. Additionally, the same species (*E. coli*) has been genetically modified to produce enzymes that degrade phenylalanine, which aids in managing phenylketonuria (PKU), a rare metabolic disorder. Isabella et al. (2018) demonstrated the efficacy of this technique in preclinical models, contributing to the development of SYNB1618, a live biotherapeutic that has progressed to human clinical trials. These case studies exemplify the promising potential of genetically engineered gut microbes to sense, record, and respond therapeutically to the physiological states of their hosts and even when these approaches are based on single-strain modifications, they highlight the importance of designing modular strains that can be assembled into stable, functional consortia with synergistic roles, thereby framing single-strain edits as foundational steps toward synthetic community engineering.

## Discussion

Although several synthetic gut microbiota have been designed for the murine model by combining meta-analytics of gut microbiota assisted by bioinformatics and culturing approaches, a common denominator is that they all depend on an assembly from collections of individual bacteria previously isolated and purified from gut samples of a model animal (Vazquez-Castellanos et al., 2019). In such an interdisciplinary approach, synthetic biology can help achieve that these microorganisms can synthesize metabolites of biological importance. Thus, therapeutic applications can be extended by genetically engineering a set of microbes. Genetically modifying synthetic gut microbiota could offer several intriguing benefits, including 1. Disease prevention and management: this approach allows for targeted therapy using engineered microbes that can produce specific compounds or outcompete harmful bacteria; 2. Enhanced digestion and nutrient absorption: altered microbes could efficiently break down nutrients and fiber, improving overall digestion; 3. Production of therapeutic compounds: modified microbes can produce compounds such as vitamins, amino acids, and anti-inflammatory agents; 4. Mental health treatment: by balancing hormones and neurotransmitters, some engineered microbes could hypothetically alleviate mental health issues like depression and anxiety; 5. Personalized medicine: this hypothetical approach may enable the introduction of specialized microbes tailored to perform specific functions for particular conditions. Additional hypothetical applications of genetically modified synthetic gut microbiota are mentioned in Table 1.

**Table 1:** Hypothetical applications of genetically modified synthetic gut microbiota.

**Area**	**Hypothetical application**	**Expected benefits**
Dysbiosis treatment	Design of gut microbiota compatible with the human colon	Colon mucosa recolonization after dysbiosis caused by disease, chemicals, or stress
Personalized therapy	Design of patient-specific microbiotas based on genome and basal microbiome	Tailored treatments for ulcerative colitis, Crohn's disease, or cancer
Therapeutic vehicle	Engineered microbes to produce and release drugs directly in the gut	Controlled release of insulin, antibodies, interleukins, or digestive enzymes
Immune modulation	Stimulating or suppressing immune responses	Prevention of autoimmune diseases or enhancement of cancer immunotherapies
Neuropsychiatry	Modulation of the gut-brain axis via microbial metabolites	Reduction of symptoms in depression, anxiety, autism, or Parkinson's disease
Metabolic diseases	Management of obesity, type 2 diabetes, and metabolic syndrome	Production of SCFAs, reduction of pro-inflammatory LPS, and improved insulin sensitivity
Infection prevention	Synthetic microbiota competing or inhibiting pathogens	Prevention of microbial pathogen infections
Intestinal detoxification	Degradation of endogenous toxins or xenobiotics	Metabolism of ammonia, oxalate, or toxic drugs like irinotecan
Oncological therapies	Use of strains that activate local immune responses or deliver anti-tumor agents	Support for anti-PD-1/PD-L1 immunotherapy in colorectal cancer or melanoma
Personalized prevention	Preventive microbiota for high-risk individuals (e.g., newborns or transplant patients)	Microbiota designed to prevent dysbiosis in premature infants or immunocompromised patients

Bacteria and yeast, including lactic acid bacteria (LAB) and *Saccharomyces cerevisiae*, two human microbiota commensals, are the most studied microbes in microbial engineering (Mahdizade Ari et al., 2024). Lactic acid bacteria, including human probiotics, are suitable chassis microbes for genetic engineering in therapy. They have been engineered for antibacterial/antiviral functions and cancer treatment by delivering “cancer vaccines” and providing defense against carcinogenesis and oxidative damage in the gastrointestinal tract (Mugwanda et al., 2023). Also, metabolic capabilities have been induced, particularly for diabetes and obesity therapy (Agarwal et al., 2014; Duan et al., 2015; Namai et al., 2018).

In this regard, the murine and human microbiota share similar phyla in their gut microbes, with Bacteroidetes and Firmicutes as dominant groups (Hugenholtz and De Vos, 2018; Sweeney and Morton, 2013). Notably, the CRISPR-Cas system is commonly detected in lactobacilli belonging to the Firmicutes phylum (Goh and Barrangou, 2019). Therefore, considering the successful genetic manipulation of probiotics and subject to verification that the CRISPR-Cas system is found in the collection of bacteria used to construct a synthetic microbiota, the group of lactobacilli could be a good starting point for engineering. However, despite the potential technical feasibility of this strategy and the advent of improved gene editing systems, it is essential to observe in gnotobiotic models how the modified bacteria behave. This approach can detect whether the modification gives them a competitive advantage over the rest, resulting in undesirable or harmful dominance and, therefore, causing dysbiosis or other adverse effects.

**Figure 1:**
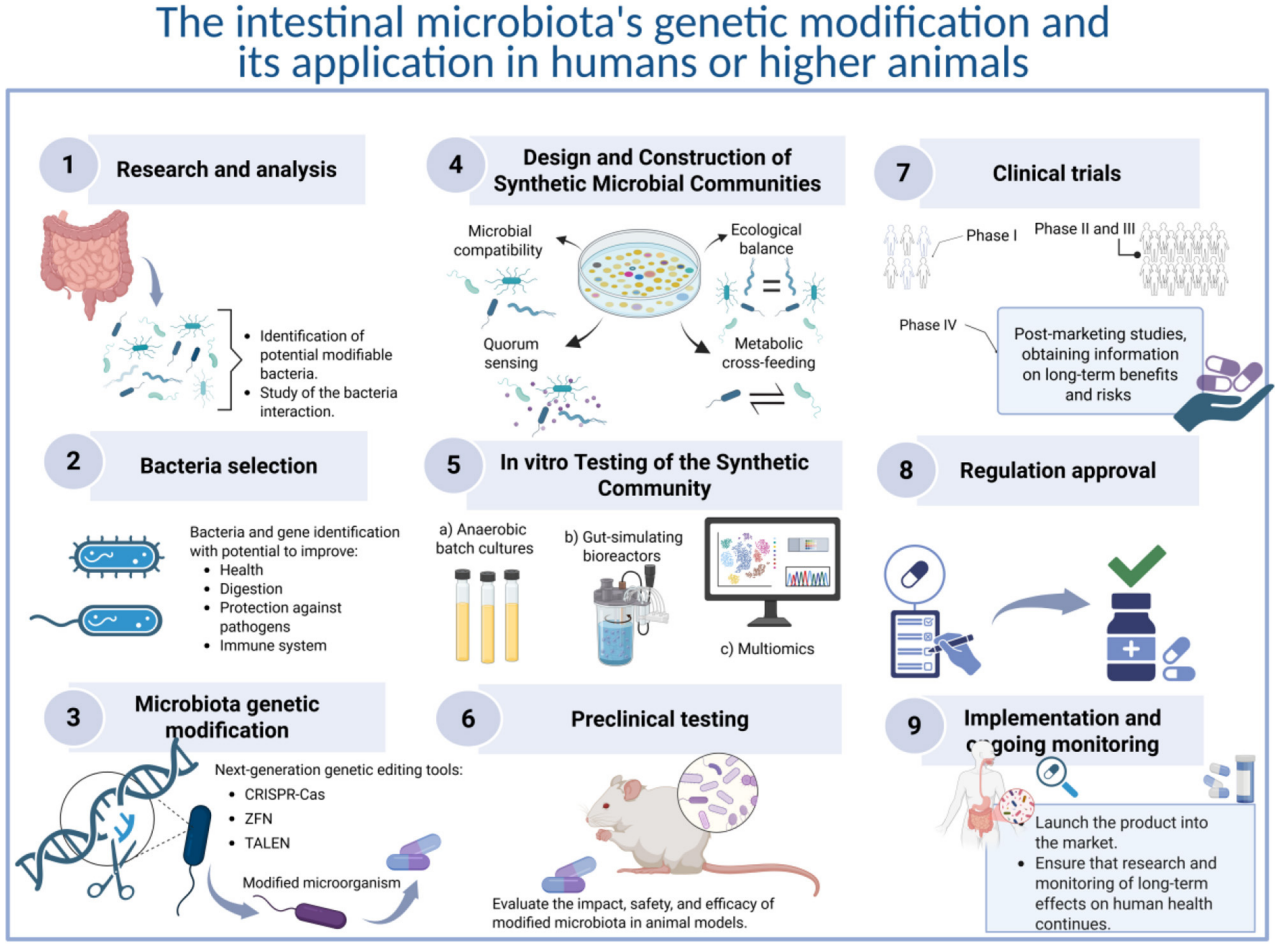
Proposed protocol for genetic modification of the intestinal microbiota and its application in humans or higher animals. 1. Research and analysis: examine the composition of the intestinal microbiota in both healthy and ill individuals to identify bacteria that could be modified to improve capabilities. Investigate the interactions between intestinal bacteria and the host to gain insights into the potential effects of these modifications. 2. Selection of bacteria for genetic manipulation: identify the specific bacteria and genes that can be targeted to modify desired functions. These functions may include improving digestion, enhancing brain development, protecting against pathogens, influencing the immune system, or any other function of interest. 3. Genetic modification: utilize genetic editing tools, primarily CRISPR-Cas9 and similar technologies, to modify the genes of the selected bacteria. This technology enables precise cuts in DNA and allows for the modification of genetic sequences with a reduced risk of losing the altered genetic material. 4. Design and construction of synthetic microbiota community: evaluate microbial compatibility, metabolic cross-feeding, quorum sensing, and ecological balance using *in vitro* co-culture systems and metabolic modeling. 5. *In vitro* testing of the synthetic community: test the community's stability, robustness, and function using gut-simulating bioreactors or anaerobic batch cultures. Use multiomics to validate functionality and emergent properties. 6. Preclinical testing: the modified bacteria's safety and efficacy must be tested in animal models, focusing on how these changes affect the overall intestinal microbiota and animal health. 7. Clinical trial phase: if animal testing yields positive results, human clinical trials can proceed, which are categorized into several phases: phase I focuses on assessing safety in a small group of participants; Phase II aims to evaluate efficacy in a larger cohort while continuing safety monitoring; Phase III is designed to confirm efficacy, observe side effects, and compare the treatment with existing options in an even larger group; and Phase IV involves post-marketing studies to collect additional information regarding long-term risks and benefits. 8. Regulatory approval: get authorization from regulatory agencies to make sure that the product is safe and effective for human use. 9. Implementation and continuous monitoring: launch the developed product into the market, ensuring it is used according to the approved indications. Continuous research and monitoring of its long-term effects on human health will be necessary.

Bacteria detected in the gut microbiota with characteristics of chassis cells should be detected and tested (Figure 1). Genetic engineering should be carried out only in gut commensals since adding non-commensal GEB carries a high risk of failure and danger due to issues of biological incompatibility. In this regard, genetic modification of the gut microbiota presents both potential benefits and risks. On the benefit side, it may offer new avenues for treating diseases, enhancing digestive health, producing beneficial substances, promoting early host development, and regulating the immune system. However, there are significant challenges and risks associated with such modifications. A primary concern is the stability of the introduced genetic changes, as microbes can quickly evolve and potentially revert to their previous states. Additionally, horizontal gene transfer raises serious issues, as modified genes might share their traits with other microbial species or even host cells, leading to unpredictable consequences. Moreover, unintended ecological impacts may emerge, disrupting the intricate communities within the gut or harming host health in unforeseen ways. However, there are specific challenges in engineering synthetic microbial communities. This includes factors like ecological stability, competition between strains, spatial structuring, quorum sensing, and horizontal gene transfer. Also, concatenating methodologies for community profiling (*e.g.*, 16S rRNA sequencing, metagenomics, and metabolic network modeling) is critical for validating synthetic microbiota functionality and interactions *in vivo*.

Although information about the risks of genetically modifying a synthetic gut microbiota is scarce, case studies of engineered probiotics could serve as a warning. For instance, in the PROPATRIA trial, patients suffering from severe acute pancreatitis who were administered a multispecies probiotic experienced a higher mortality rate compared to the placebo group, indicating potential risks in critically ill patients (Besselink et al., 2004). Furthermore, there have been rare but documented instances of sepsis and bacteremia linked to probiotic strains such as *Lactobacillus rhamnosus* in immunocompromised individuals (Salminen et al., 2004). Additional concerns stem from the potential for gene transfer; for example, a study registered antimicrobial resistance genes in commercial probiotic products intended for animals, some of which were located on plasmids, heightening the risk of horizontal gene transfer (Tóth et al., 2021). These findings emphasize the need for rigorous safety evaluations of genetically modified probiotics and synthetic gut microbiota.

Ethical considerations are central to the development of genetically modified microbiota, requiring a strong focus on safety, informed consent, and equitable access. Equally important is the creation of robust regulatory frameworks, including standardized risk assessments, post-deployment monitoring, and international collaboration to ensure consistency and safety across borders. Future research should prioritize long-term studies to evaluate the effects of engineered microbes on host health and microbiota dynamics. Multi-omics approaches can offer deeper insights into microbial interactions and safety profiles. Investment in biosafety features like kill switches and refined gene-editing tools is also essential.

A structured roadmap is advised: (1) validation through laboratory and animal models, (2) ethically approved pilot clinical trials, (3) open-access data sharing to ensure transparency, and (4) ongoing refinement of regulatory guidelines. Engaging the public and providing education will be essential in building trust and promoting responsible innovation. By aligning scientific research, policy, and ethical considerations, we can safely advance the application of genetically modified probiotics in medicine and biotechnology.

## References

[B1] Agarwal P. Khatri P. Billack B. Low W.-K. Shao J. (2014). Oral delivery of glucagon like peptide-1 by a recombinant Lactococcus lactis. Pharm. Res. 31, 3404–3414. 10.1007/s11095-014-1430-3 24928365

[B2] Allemailem K. S. Almatroudi A. Rahmani A. H. Alrumaihi F. Alradhi A. E. Alsubaiyel A. M.. (2024). Recent updates of the CRISPR/Cas9 genome editing system: novel approaches to regulate its spatiotemporal control by genetic and physicochemical strategies. Int. J. Nanomedicine 6, 5335–5363. 10.2147/IJN.S455574 38859956 PMC11164216

[B3] Besselink M. G. Timmerman H. M. Buskens E. Nieuwenhuijs V. B. Akkermans L. M. Gooszen H. G. (2004). Probiotic prophylaxis in patients with predicted severe acute pancreatitis (PROPATRIA): design and rationale of a double-blind, placebo-controlled randomised multicenter trial [ISRCTN38327949]. BMC Surgery 4, 1–7. 10.1186/1471-2482-4-12 15456517 PMC526218

[B4] Chan C. T. Lee J. W. Cameron D. E. Bashor C. J. Collins J. J. (2016). 'Deadman'and‘Passcode'microbial kill switches for bacterial containment. Nat. Chem. Biol. 12, 82–86. 10.1038/nchembio.1979 26641934 PMC4718764

[B5] Duan F. F. Liu J. H. March J. C. (2015). Engineered commensal bacteria reprogram intestinal cells into glucose-responsive insulin-secreting cells for the treatment of diabetes. Diabetes 64, 1794–1803. 10.2337/db14-0635 25626737 PMC4407861

[B6] Durmusoglu D. Al'abri I. S. Collins S. P. Cheng J. Eroglu A. Beisel C. L.. (2021). *In situ* biomanufacturing of small molecules in the mammalian gut by probiotic *Saccharomyces boulardii*. ACS Synth. Biol. 10, 1039–1052. 10.1021/acssynbio.0c00562 33843197 PMC12977008

[B7] Goh Y. J. Barrangou R. (2019). Harnessing CRISPR-cas systems for precision engineering of designer probiotic lactobacilli. Curr. Opin. Biotechnol. 56, 163–171. 10.1016/j.copbio.2018.11.009 30530241

[B8] Hallet B. Sherratt D. J. (1997). Transposition and site-specific recombination: adapting DNA cut-and-paste mechanisms to a variety of genetic rearrangements. FEMS Microbiol. Rev. 21, 157–178. 10.1016/S0168-6445(97)00055-7 9348666

[B9] Hugenholtz F. De Vos W. M. (2018). Mouse models for human intestinal microbiota research: a critical evaluation. Cell. Mol. Life Sci. 75, 149–160. 10.1007/s00018-017-2693-8 29124307 PMC5752736

[B10] Isabella V. M. Ha B. N. Castillo M. J. Lubkowicz D. J. Rowe S. E. Millet Y. A.. (2018). Development of a synthetic live bacterial therapeutic for the human metabolic disease phenylketonuria. Nat. Biotechnol. 36, 857–864. 10.1038/nbt.4222 30102294

[B11] Jiang F. Doudna J. A. (2017). CRISPR–Cas9 structures and mechanisms. Annu. Rev. Biophys. 46, 505–529. 10.1146/annurev-biophys-062215-010822 28375731

[B12] Jiang X. Yan C. Zhang H. Chen L. Jiang R. Zheng K.. (2023). Oral probiotic expressing human ethanol dehydrogenase attenuates damage caused by acute alcohol consumption in mice. Microbiol. Spectr. 11, e04294–e04222. 10.1128/spectrum.04294-22 37039510 PMC10269551

[B13] Li C. Han Y. Zou X. Zhang X. Ran Q. Dong C. (2024). A systematic discussion and comparison of the construction methods of synthetic microbial community. Synth. Syst. Biotechnol. 9, 775–783. 10.1016/j.synbio.2024.06.006 39021362 PMC11253132

[B14] Liu Y. Feng J. Pan H. Zhang X. Zhang Y. (2022). Genetically engineered bacterium: principles, practices, and prospects. Front. Microbiol. 13:997587. 10.3389/fmicb.2022.997587 36312915 PMC9606703

[B15] Mahdizade Ari M. Dadgar L. Elahi Z. Ghanavati R. Taheri B. (2024). Genetically engineered microorganisms and their impact on human health. Int. J. Clin. Pract. 2024:6638269. 10.1155/2024/6638269 38495751 PMC10944348

[B16] Mazhar S. F. Afzal M. Almatroudi A. Munir S. Ashfaq U. A. Rasool M.. (2020). The prospects for the therapeutic implications of genetically engineered probiotics. J. Food Qual. 2020:9676452. 10.1155/2020/9676452

[B17] Mooser C. De Agüero M. G. Ganal-Vonarburg S. C. (2018). Standardization in host–microbiota interaction studies: challenges, gnotobiology as a tool, and perspective. Curr. Opin. Microbiol. 44, 50–60. 10.1016/j.mib.2018.07.007 30056329

[B18] Mugwanda K. Hamese S. Van Zyl W. F. Prinsloo E. Du Plessis M. Dicks L. M.. (2023). Recent advances in genetic tools for engineering probiotic lactic acid bacteria. Biosci. Rep. 43:BSR20211299. 10.1042/BSR20211299 36597861 PMC9842951

[B19] Namai F. Shigemori S. Sudo K. Sato T. Yamamoto Y. Nigar S.. (2018). Recombinant mouse osteocalcin secreted by *Lactococcus lactis* promotes glucagon-like peptide-1 induction in STC-1 cells. Curr. Microbiol. 75, 92–98. 10.1007/s00284-017-1354-3 28905106

[B20] Nazir A. Hussain F. H. N. Raza A. (2024). Advancing microbiota therapeutics: the role of synthetic biology in engineering microbial communities for precision medicine. Front. Bioeng. Biotechnol. 12:1511149. 10.3389/fbioe.2024.1511149 39698189 PMC11652149

[B21] Porteus M. H. Carroll D. (2005). Gene targeting using zinc finger nucleases. Nat. Biotechnol. 23, 967–973. 10.1038/nbt1125 16082368

[B22] Riglar D. T. Giessen T. W. Baym M. Kerns S. J. Niederhuber M. J. Bronson R. T.. (2017). Engineered bacteria can function in the mammalian gut long-term as live diagnostics of inflammation. Nat. Biotechnol. 35, 653–658. 10.1038/nbt.3879 28553941 PMC5658125

[B23] Romero-Luna H. E. Hernández-Mendoza A. González-Córdova A. F. Peredo-Lovillo A. (2022). Bioactive peptides produced by engineered probiotics and other food-grade bacteria: a review. Food Chemistry X 13:100196. 10.1016/j.fochx.2021.100196 35498967 PMC9039921

[B24] Salminen M. K. Rautelin H. Tynkkynen S. Poussa T. Saxelin M. Valtonen V.. (2004). Lactobacillus bacteremia, clinical significance, and patient outcome, with special focus on probiotic L. rhamnosus GG. Clin. Infect. Dis. 38, 62–69. 10.1086/380455 14679449

[B25] Sana T. G. Laubier A. Bleves S. (2014). Gene transfer: conjugation. Pseudomonas: Methods Protoc. 1149, 17–22. 10.1007/978-1-4939-0473-0_3 24818893

[B26] Shizuya H. Kouros-Mehr H. (2001). The development and applications of the bacterial artificial chromosome cloning system. Keio J. Med. 50, 26–30. 10.2302/kjm.50.26 11296661

[B27] Sun N. Zhao H. (2013). Transcription activator-like effector nucleases (TALENs): a highly efficient and versatile tool for genome editing. Biotechnol. Bioeng. 110, 1811–1821. 10.1002/bit.24890 23508559

[B28] Sweeney T. E. Morton J. M. (2013). The human gut microbiome: a review of the effect of obesity and surgically induced weight loss. JAMA Surg. 148, 563–569. 10.1001/jamasurg.2013.5 23571517 PMC4392891

[B29] Tóth A. G. Csabai I. Judge M. F. Maróti G. Becsei Á. Spisák S.. (2021). Mobile antimicrobial resistance genes in probiotics. Antibiotics 10:1287. 10.3390/antibiotics10111287 34827225 PMC8614787

[B30] Vazquez-Castellanos J. F. Biclot A. Vrancken G. Huys G. R. Raes J. (2019). Design of synthetic microbial consortia for gut microbiota modulation. Curr. Opin. Pharmacol. 49, 52–59. 10.1016/j.coph.2019.07.005 31430629

[B31] Xin Y. Qiao M. (2025). Towards microbial consortia in fermented foods for metabolic engineering and synthetic biology. Food Res. Int. 201:115677. 10.1016/j.foodres.2025.115677 39849795

